# Prevalence of *T. rubrum* and *T. interdigitale* Exhibiting High MICs to Terbinafine in Clinical Samples Analyzed in the Portuguese Mycology Reference Laboratory

**DOI:** 10.3390/pathogens14020115

**Published:** 2025-01-25

**Authors:** Helena Schirmer, Camila Henriques, Helena Simões, Cristina Veríssimo, Raquel Sabino

**Affiliations:** 1 Department of Basics Health Sciences, Federal University of Health and Sciences of Porto Alegre (UFCSPA), Porto Alegre 90050-170, Brazil; 2 National Reference Laboratory for Influenza and Other Respiratory Virus, National Institute of Health Doutor Ricardo Jorge (INSA), 1649-016 Lisbon, Portugal; camila.henriques@insa.min-saude.pt; 3 Reference Unit for Parasitic and Fungal Infections, Department of Infectious Diseases, National Institute of Health Doutor Ricardo Jorge (INSA), 1649-016 Lisbon, Portugal; helena.simoes@insa.min-saude.pt (H.S.); cristina.verissimo@insa.min-saude.pt (C.V.); 4 Faculdade de Farmácia, Universidade de Lisboa, 1649-003 Lisbon, Portugal; raquel.sabino@ff.ulisboa.pt; 5 Faculdade de Medicina, Instituto de Saúde Ambiental, Universidade de Lisboa, 1649-028 Lisbon, Portugal; 6 Laboratório Associado TERRA—Laboratório para o Uso Sustentável da Terra e dos Serviços dos Ecossistemas, Instituto Superior de Agronomia, 1349-017 Lisbon, Portugal

**Keywords:** *Trichophyton*, antifungal resistance, dermatophytes, squalene epoxidase mutations, terbinafine

## Abstract

Cutaneous fungal infections represent a significant burden worldwide with a high impact on public health. Accurate identification of dermatophyte species causing these infections is vital for an appropriate treatment. Terbinafine is the primary agent against *Trichophyton* species due to its clinical efficacy; however, cases of elevated minimum inhibitory concentration (MIC) have been reported, raising clinical and epidemiological concerns. Herein, we aimed to detect *Trichophyton rubrum* and *Trichophyton interdigitale* isolates collected from clinical samples with terbinafine-high MICs (TRB-hMIC). A total of 168 isolates, recovered from 2017 to 2023, were identified as *T. rubrum* complex (140/83.4%) or *T. interdigitale* (28/16.7%) and further screened regarding their terbinafine susceptibility. Four isolates with capacity to grow in terbinafine media were detected by screening, and these and a further sixteen random isolates were submitted to the broth microdilution method. This methodology confirmed the four (2.4%) isolates as TRB-hMIC. One *T. rubrum* and three *T. interdigitale* showed a minimum inhibitory concentration (MIC) higher than 1 mg/L. Partial sequencing of the SQLE gene identified point mutations in *T. rubrum* (Phe397Iso) and in one *T. interdigitale* (Phe397Leu) isolate. Notably, in the other two *T. interdigitale* isolates with TRB-hMIC, no point mutations in the SQLE gene were identified. In conclusion, TRB-hMIC isolates (*T. rubrum* and *T. interdigitale*) were identified in clinical samples analyzed in Portugal, as antifungal susceptibility testing is a crucial routine for identifying treatment failures and also for epidemiological purposes aiming to monitor the dynamics of terbinafine resistance.

## 1. Introduction

Nearly a billion people are estimated to have skin, nail, and hair fungal infections [1], and this condition is the most common cause of skin disease in the European population [2]. In the United States, these infections represent more than eight million outpatient visits and result in healthcare system costs exceeding USD 2.4 billion, with USD 820 million dedicated to treating dermatophyte infections [3]. 

The most prevalent worldwide dermatophyte species are *Trichophyton rubrum* and *T. interdigitale* [4,5,6,7]. Accurate dermatophyte identification at the species level is important for both identification of the source of infection and appropriate treatment. Terbinafine is the first-line antifungal agent against *Trichophyton* species due to its clinical efficacy as continuous or as pulse therapy [8,9]. Although dermatophytes are rarely resistant to terbinafine, some *T. rubrum* and *T. interdigitale* isolates showing resistance to this antifungal have been identified around the world [10,11,12,13]. Reports of recalcitrant infections caused by a *Trichophyton* isolate, initially described as *T. mentagrophytes* VIII and currently identified as *T. Indotineae*, raised attention on terbinafine resistance. This isolate was associated with cases of dermatophytosis in India, but terbinafine resistance is already spreading to other continents and countries [14,15,16].

Terbinafine is an antifungal belonging to the allylamines class, whose mechanism of action is the inhibition of squalene epoxidase (SQLE), an enzyme involved in the early steps of ergosterol biosynthesis. The accumulation of squalene is toxic to the fungal cell [12]. Point mutations in the open reading frame (ORF) of the SQLE gene that lead to amino acid substitutions within the SQLE protein (Leu^393^, Phe^397^, Phe^415^, and His^440^) are associated with terbinafine resistance in *Trichophyton* spp. [17]. These amino acid substitutions negatively affect the affinity of the enzyme to the antifungal agent. 

The gold-standard method to determine the antifungal susceptibility profile is broth microdilution [18,19]. However, this procedure is not usually performed routinely because it is laborious and time-consuming, and the chance of microbiological contamination is high. Therefore, antifungal resistance, including resistance to terbinafine in dermatophytes, is underestimated. Some studies have used a screening technique with agar supplemented with terbinafine as a feasible alternative to initial detection in the laboratory routine of possible resistance [12,20]. No clinical breakpoints were established by the Clinical & Laboratory Standards Institute (CLSI) or European Committee on Antimicrobial Susceptibility Test (EUCAST) to *T. rubrum* and *T. interdigitale* for terbinafine. However, in 2020, the EUCAST suggested a terbinafine concentration of 0.03 mg/L as the epidemiological cut-off value (ECOFF) to *T. rubrum* [18]. Additionally, a multicenter study proposed that strains belonging to the *T. rubrum* complex and *T. interdigitale* isolates presenting a minimum inhibitory concentration (MIC) of 0.03 mg/L and 0.125 mg/L, respectively [20], could be classified as wild-type strains.

Given the lack of epidemiological data concerning *Trichophyton* infections with high MIC to terbinafine, the aim of this study was to identify isolates of *T. rubrum* and *T. interdigitale* from clinical samples analyzed in the Portuguese Mycology Reference Laboratory and detect possible TRB-hMIC isolates by screening method, microdilution, and molecular approaches. 

## 2. Materials and Methods 

### 2.1. Isolates

Samples from keratinized tissue were collected from patients with suspicion of fungal infection who were assisted at the National Institute of Health Dr. Ricardo Jorge (INSA, Lisbon, Portugal) or from particular clinics, hospitals, or other health centers assisted by the Portuguese Mycology Reference Laboratory at INSA. Data collection was performed between 2017 and 2023. Direct microscopic examination was performed on all samples. In addition, samples (skin, nail scrapings, and hairs) were cultured on Sabouraud Dextrose Agar (SDA) supplemented with chloramphenicol (Difco, Detroit, MI, USA) and Mycosel agar medium (Difco, Detroit, MI, USA). For species identification, conventional methods were performed, namely, macro- and microscopic identification and a urease probe whenever needed. MALDI-TOF based identification and/or PCR followed by ITS and calmodulin sequencing were performed on almost all samples. Only the isolates identified as *T. rubrum* complex or *T. interdigitale* were then screened for antifungal susceptibility to terbinafine. To characterize the samples enrolled in this study, some information was obtained from the clinical request, such as age and gender of the patient as well as the site of the lesion. No information could be obtained regarding patients’ travel history or previous antifungal treatments.

### 2.2. Terbinafine Resistance Screening

Screening media were used to analyze the capacity for growth in the presence of terbinafine of all isolates belonging to the *T. rubrum* complex and *T. interdigitale*. A spore suspension of each isolate, corresponding to 0.5 McFarland, was inoculated with a swab onto both an SDA plate supplemented with terbinafine (0.06 mg/mL, 0.125 mg/mL, or 0.03 mg/mL) and without chloramphenicol and onto an SDA plate without antifungal added (to serve as growth control). The plates were then incubated at 27 °C for 15 days, and the growth was analyzed on days #7, #10, and #15. For the isolates collected in the period 2017 to 2020, the screening was performed using two terbinafine concentrations: 0.06 mg/L and 0.125 mg/L. For isolates recovered from 2022 to 2023, the concentration used was 0.03 mg/L, according to the ECOFF supported by EUCAST, published in the meantime [18]. The isolates that grew in both the control medium and the medium with terbinafine were then further studied for the determination of the MIC by microdilution broth.

### 2.3. Determination of Minimum Inhibitory Concentration (MIC) to Terbinafine

All isolates that grew on the screening media and 16 isolates that did not grow on the screening media and that were randomly selected were then submitted for MIC determination following the methodology standardized by CLSI, document M38 [19]. To determine the MIC, an inoculum suspension was prepared on RPMI medium and then inoculated into the microplates with a serial dilution of terbinafine (0.0156 to 8 mg/L). As controls, a sterile control (only medium) and a growth control (without antifungal) were also included.

An internal control strain (*Aspergillus flavus* ATCC 204304) with known susceptibility (MIC to terbinafine between 0.25 and 1 mg/L) was included as a positive control for MIC determination. Microplates were incubated at 25 °C for 5–7 days, and the results were read on day #7; the MIC corresponded to the first antifungal concentration reduction in 80% of growth when compared with the growth control.

### 2.4. Amplification and Sequencing of the SQLE Gene in T. rubrum Complex and T. interdigitale Isolates

To perform the partial sequencing of the SQLE gene, DNA was extracted from fresh isolates using a High Pure PCR Template Preparation Kit (Roche Diagnostics GmbH, Mannheim, Germany) according to the manufacturer’s instructions. To identify point mutations at the SQLE gene, a PCR reaction was performed using the set of primers Drsq1 and Drsq2, as described by Zahra et al. [21]. The final volume was 25 μL, as follows: 1.5 μL of forward and reverse primers (10 uM), 18 μL of ultrapure water, 1 bead of illustraTM puReTaq Ready-To-GoTM PCR Beads (GE Healthcare Life, Chicago, IL, USA), and 4 μL of DNA. Regarding the sequencing reaction, the final volume was 10 μL, as follows: 2 μL of each primer (10 uM), 3 μL of ultrapure water, 1 μL of BigDye® (ThermoFisher Scientific, Waltham, MA, USA), 3 μL of buffer, and 1 μL of the PCR product. The consensus sequences were obtained with the Chromas Lite program, and point mutation analysis was performed using the software MEGA7 (Molecular Evolutionary Genetics Analysis version 7.0). The GenBank accession numbers corresponding to the sequences of the ITS region and of the partial sequences of calmodulin and SQLE genes are described in Table 1. These sequences were obtained from the isolates identified as TRB-hMIC (#59, 63, 94, 126) and also from randomly selected susceptible isolates (#41, 49, 65, 143). Resistant and susceptible *T. rubrum* and *T. interdigitale* isolates were aligned with a susceptible and a resistant reference sequence. Reference sequences used were the following: GenBank accession numbers for *T. rubrum*: Ay282412.1 (resistant) and MG587093.2 (susceptible); for *T. interdigitale*: MH114933.1 (resistant) and MN893286.1 (susceptible). 

## 3. Results

### 3.1. Population Characteristics

The characteristics of the studied population are described in Table 2. The median age was 45 years old (ranging between 3 and 81 years), with individuals aged 41 to 50 years (14.3%) being the most affected, followed by the group of 61 to 70 years (13.1%). Regarding the gender, 91 patients (54.2%) were male, 64 (38.1%) were female, and in 13 (7.7%) of the cases, the gender was not mentioned. Nails (N = 127; 75.6%) were the most common biological sample sent to the laboratory with a suspicion of dermatophytosis, followed by glabrous skin (N = 25; 14.9%). In the case of three patients, nail and skin samples were both sent.

### 3.2. Isolates

From 2017 to 2023, a total of 224 isolates were identified as *T. rubrum* complex or *T. interdigitale* and were selected for the terbinafine screening test. In the process of obtaining a fresh culture from our frozen collection in order to perform the terbinafine screening test, we were not able to recover 50 isolates given the loss of viability of the culture. In the end, 168 isolates were submitted to the terbinafine screening test. From those, 119 isolates were obtained from 2017 to 2020, and 49 isolates were obtained from 2022 to 2023. From the 168 isolates, 76.8% were identified as *T. rubrum*, 6.5% *T. violaceum*, and 16.7% *T. interdigitale*. The *T. interdigitale* isolates identified as non-wild type to terbinafine were then submitted to sequencing in order to identify if any *T. indotineae* isolate was present. All isolates were confirmed to be *T. interdigitale*. 

### 3.3. Terbinafine Susceptibility 

**Figure 1:**
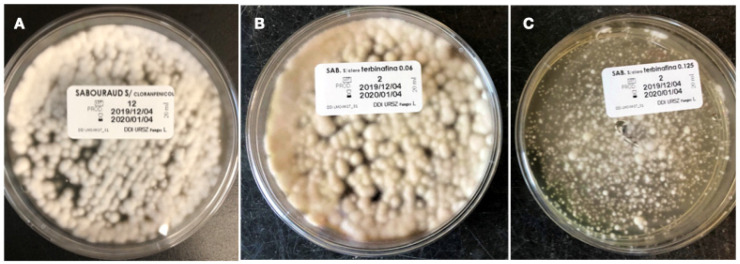
Growth of the *T. rubrum* isolate #126 (MIC = 8 mg/L), resistant to terbinafine in the terbinafine screening agar. (**A**) = growth control; (**B**) = SDA with terbinafine 0.06 mg/L; (**C**) = SDA with terbinafine 0.125 mg/L.

**Figure 2:**
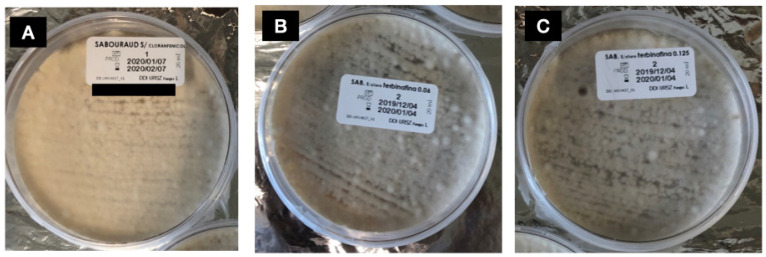
Growth of *T. interdigitale* isolate #94 (MIC > 8 mg/L), resistant to terbinafine in the terbinafine screening agar. (**A**) = growth control; (**B**) = SDA with terbinafine 0.125 mg/L; (**C**) = SDA with terbinafine 0.06 mg/L.

The total number of isolates (168) were tested for the terbinafine screening, and growth was observed in media supplemented with terbinafine in both concentrations (0.06 μg/mL and 0.125 μg/mL) in 4 (2.4%) isolates. The isolates with capacity to grow in terbinafine using the screening method were one *T. rubrum* (Figure 1) and three *T. interdigitale* (Figure 2). Three of those isolates were collected from nail scrapings (*T. interdigitale* #59, #63 and *T. rubrum* #126) and one from glabrous skin (*T. interdigitale* #94) (Table 2). 

To confirm this result and to establish the MIC, broth microdilution was performed to those four isolates and to sixteen other susceptible isolates. The results of both procedures were in concordance since the MICs for those four isolates were high (>1 mg/L) (Table 3). 

### 3.4. SQLE Gene Sequencing

To understand the mechanism of resistance to terbinafine, partial sequencing of the SQLE gene was performed in the four isolates showing TRB-hMIC. Single-nucleotide polymorphisms (SNPs) resulting in missense mutations were identified in only two isolates: one *T. rubrum* (isolate #126) and one *T. interdigitale* (isolate #94). In both isolates, the SNP was detected at position 1189 in the ORF of the SQLE gene. In the *T. rubrum* isolate, this mutation resulted in the substitution of the amino acid Phenylalanine into an Isoleucine (Phe397Iso) and in the *T. interdigitale* isolate, the mutation corresponded to a substitution of the amino acid Phenylalanine into a Leucine (Phe397Leu). In the other two isolates, none of the most common known point mutations in the SQLE gene were detected.

## 4. Discussion

Although some reports have identified dermatophytes with a high MIC to terbinafine, the real prevalence of the resistance to this antifungal is still underestimated in the practical routine since antifungal susceptibility tests are not performed regularly. Even when the prevalence of *Trichophyton* isolates resistant to terbinafine is low, the proper species identification and further susceptibility testing are crucial to obtain epidemiological data and to proceed to an adequate treatment, avoiding unnecessary costs to the patient and to healthcare systems. 

The prevalence of TRB-hMIC *Trichophyton* species is variable worldwide and varies between 0.5% and 18%, but it may be higher (around 70%), for example, in India, where the presence of *T. indotineae* is more common [12,22,23]. In our study, the prevalence of *Trichophyton* isolates of the non-wild type for terbinafine was 2.4% (one *T. rubrum* and three *T. interdigitale*). A similar prevalence was reported by Yamada et al. [12], although the majority of the non-wild-type isolates from that study was identified as *T. rubrum*. In a cohort study from Lausanne, the authors analyzed all *Trichophyton* isolates collected during a period of 8 years, and the prevalence of TRB resistance in those isolates was 0.83%, with an increased detection of isolates with growth capacity in TRB from 2013 (0.63%) to 2021 (1.3%) [22]. In a French study, the reported prevalence of TRB-hMIC was 0.5%, and as in the previously mentioned study, only one isolate of *T. indotineae* was identified [24].

On the other hand, in North America, the prevalence of terbinafine-resistant *Trichophyton* isolates was around 19%, and the most prevalent species were the ones from the *T. rubrum* complex followed by the *T. mentagrophytes* complex and *T. indotineae* [25]. When we compared our results with studies from India, the prevalence of terbinafine-resistant isolates is much higher in this country, but the main etiological agent identified as resistant was *T. indotineae* [23]. Terbinafine treatment failure is more frequently observed in infections with *T. indotineae*, a species included in the *T. mentagrophytes* complex; however, this species has virulence and resistance profiles different from other species belonging to the same complex. As *T. indotineae* has been reported in different countries other than India, we sequenced our *T. interdigitale* isolates that showed resistance to terbinafine (N = 3) to be sure that we did not miss the proper identification. This procedure, together with MALDI-TOFF data, confirmed that our isolates were, in fact, *T. interdigitale* and none of them were *T. indotineae*. 

The differences in species and TRB-resistance prevalences among countries highlight the importance of incorporating species identification and susceptibility profiling into routine laboratory practice. 

In studies where isolates capable of growing in terbinafine were identified, the MICs ranged from >0.5 μg/mL to >12.8 μg/mL [12,25,26]. In our study, one isolate showed a MIC = 8 μg/mL, two isolates a MIC >8 μg/mL and one a MIC = 1 μg/mL. Yamada et al. [12] reported a high variability of MIC values obtained in their study; this difference was associated with the detected SQLE gene mutations. Higher MIC values were related to Phe397Leu and Leu393Phe substitutions. 

In 2020, the proposed ECOFF for *T. rubrum* was 0.03 μg/mL [18]. For *T. interdigitale*, the suggested ECOFF was 0.125 μg/mL [20]. During the period 2017–2020, we prepared the screening media by supplementing the SDA agar with terbinafine with the following concentrations: 0.06 mg/L and 0.125 mg/L, as initially was recommended by Saunte et al. [27]. After that period, and following the most recent recommendations [18,20], we switched the terbinafine concentration to 0.03 and 0.125. This modification in the procedure did not seem to affect the detection of resistant isolates to terbinafine in the first period of our study, since all resistant isolates grew in the highest concentration as well. 

The most prevalent point mutations associated with elevated MIC to terbinafine are Leu393Phe and Phe397Leu [12]. However, others have been previously documented, such as Leu393Ser, Phe397Ile, Phe397Leu, Phe397Val, Phe415Val, and His440Tyr [12]. In our study, in one TRB-hMIC *T. interdigitale* collected from the thigh skin (#94), we identified the point mutation Phe397Leu, which was therefore correlated with the high MIC (>8 mg/L) observed. Similarly, in the *T. rubrum* isolate #126, the detected point mutation was Phe397Iso, also linked to a high MIC (8 mg/L). This isolate was collected from nail scrapings. Given the challenging nature of nail tissue treatment, particularly with topical antifungals, the possibility of the maintenance of the etiological agent and further development of resistance cannot be dismissed. Also, given the elevated number of antifungals sold without medical prescription for the treatment of onychomycosis, we cannot rule out a future rise in the number of *T. rubrum* or *T. interdigitale* isolates resistant to terbinafine. 

Since no information could be obtained regarding the patients’ travel history or previous antifungal treatment with terbinafine, it is not possible to correlate those data with the obtained results, which represents a limitation of our findings.

Interestingly, in two isolates with high MICs, no point mutations were detected in the SQLE gene. This observation aligns with findings from other studies and suggests that the absence of point mutations associated with high MICs may indicate the existence of alternative mechanisms conferring in vitro terbinafine resistance [27,28]. Furthermore, we performed the partial sequencing of the SQLE gene. Although we selected the region where the majority of the TRB-resistance-associated mutations are described, we cannot rule out the existence of mutations in other regions of the SQLE gene, which can be pointed out as a limitation of our study. Additionally, we aligned our sequences with only one reference strain to each case, which may have led to the loss of some information that greater variability could have provided. It is plausible that the complete gene sequencing is warranted to comprehensively identify point mutations within the entire gene. Therefore, more studies should be conducted in those isolates in order to understand which mutations are associated with their mechanism of resistance.

Despite the increasing prevalence of TRB-hMIC dermatophytes, antifungal susceptibility testing is not routinely performed as it is labor-intensive, time-consuming, and susceptible to contamination. The agar-based method could be implemented as a screening methodology for detection of TRB-hMIC since it is reliable and easy to perform. We found 100% agreement between the results of agar-based screening and microdilution methodology using the EUCAST ECOFF value. The same result was shown by Sioppi et al. [29] to non-wild-type isolates of *Trichophyton* sp. Also, this screening method shows high sensitivity and is supported by EUCAST to easily detect antifungal resistances in other fungi, as for *Aspergillus* resistant to azoles and echinocandins [30,31,32]. 

## 5. Conclusion

Although in a low prevalence, it was possible to detect TRB-hMIC *T. rubrum* and *T. interdigitale* isolates in our study, showing that non-wild-type strains are circulating and being isolated from clinical samples in Portugal. Hence, we reinforce the importance of routinely performing susceptibility antifungal testing in order to perceive failures in treatment and also for epidemiological purposes, following the dynamics of terbinafine resistance.

## Figures and Tables

**Table 1:** GenBank accession numbers for the ITS region and the partial sequences of calmodulin and SQLE gene of *T. rubrum* and *T. interdigitale* spp. isolates.

GenBank Accession Numbers					
		Isolate	ITS	Calmodulin	SQLE
**TRB-hMIC isolates**	*T. interdigitale*	59	PQ873113	PQ876114	PQ876116
	*T. interdigitale*	63	PQ873114	PQ876115	PQ876117
	*T. interdigitale*	94	PQ867496	PQ876107	PQ876113
	*T. rubrum*	126	PQ867489	PQ876102	PQ876108
**Susceptible isolates**	*T. interdigitale*	41	PQ873112	PQ876104	PQ876110
	*T. interdigitale*	49	PQ867494	PQ876105	PQ876111
	*T. interdigitale*	65	PQ867495	PQ876106	PQ876112
	*T. rubrum*	143	PQ867490	PQ876103	PQ876109

TRB-hMIC = terbinafine-high MIC.

**Table 2:** Population characteristics and biological samples of the study from 2017 to 2023.

	N	%
**Female**	64	38.1
**Male**	91	54.2
**NR**	13	7.7
**Median age**	45	(3–81 years)
**Age range**		
Up to 10 years	12	7.1
11 to 20 years	15	8.9
21 to 30 years	14	8.3
31 to 40 years	20	11.9
41 to 50 years	25	14.9
51 to 60 years	19	11.3
61 to 70 years	22	13.1
>70 years	13	7.7
NR	28	16.7
**Biological samples**		
Nail	127	75.6
Glabrous skin	25	14.9
Scalp	1	0.6
Nail + skin	3	1.8
Others	7	4.1
NR	5	3.0

NR = not referred.

**Table 3:** Antifungal susceptibility of the studied *T. rubrum* and *T. interdigitale* isolates by screening agar method, minimum inhibitory concentration, and identification of SQLE point mutations.

		TRB Screening Media			MIC (mg/L)	
	Isolate ID (#)	0.06 mg/L	0.125 mg/L	Species	TRB	SQLE Gene
**TRB-hMIC isolates**	59	+	+	*T. interdigitale*	1	ND
	63	+	+	*T. interdigitale*	>8	ND
	94	+	+	*T. interdigitale*	>8	Phe397Leu
	126	+	+	*T. rubrum*	8	Phe397Iso
**Susceptible isolates**	51	-	-	*T. rubrum*	0.25	NP
	62	-	-	*T. rubrum*	1	NP
	68	-	-	*T. rubrum*	1	NP
	80	-	-	*T. rubrum*	1	NP
	81	-	-	*T. rubrum*	0.5	NP
	9	-	-	*T. interdigitale*	0.0625	NP
	15	-	-	*T. interdigitale*	0.25	NP
	16	-	-	*T. interdigitale*	NA *	NP
	17	-	-	*T. interdigitale*	0.25	NP
	32	-	-	*T. interdigitale*	0.25	NP
	41	-	-	*T. interdigitale*	0.25	NP
	49	-	-	*T. interdigitale*	0.25	NP
	61	-	-	*T. interdigitale*	0.25	NP
	65	-	-	*T. interdigitale*	0.25	NP
	130	-	-	*T. interdigitale*	0.25	NP
	148	-	-	*T. interdigitale*	0.25	NP

NA = not applicable, ND = not detected, NP = not performed. + = with growth, - = without growth. * loss of viability of the culture.

## Data Availability

The original contributions presented in this study are included in the article. Further inquiries can be directed to the corresponding author(s).

## Abbreviations

The following abbreviations are used in this manuscript:

CLSI—Clinical & Laboratory Standards Institute
ECOFF—epidemiological cut-off value
EUCAST—European Committee on Antimicrobial Susceptibility Test
MIC—minimum inhibitory concentration
ORF—open reading frame
SQLE—squalene epoxidase
SDA—Sabouraud Dextrose Agar
TRB—terbinafine
